# The Validation of a Portable Functional NIRS System for Assessing Mental Workload

**DOI:** 10.3390/s21113810

**Published:** 2021-05-31

**Authors:** Manob Jyoti Saikia, Walter G. Besio, Kunal Mankodiya

**Affiliations:** Department of Electrical, Computer and Biomedical Engineering, University of Rhode Island, Kingston, RI 02881, USA; besio@uri.edu (W.G.B.); kunalm@uri.edu (K.M.)

**Keywords:** fNIRS, optical sensor, near-infrared spectroscopy, working memory, cognitive load, brain imaging, prefrontal cortex

## Abstract

Portable functional near-infrared spectroscopy (fNIRS) systems have the potential to image the brain in naturalistic settings. Experimental studies are essential to validate such fNIRS systems. Working memory (WM) is a short-term active memory that is associated with the temporary storage and manipulation of information. The prefrontal cortex (PFC) brain area is involved in the processing of WM. We assessed the PFC brain during n-back WM tasks in a group of 25 college students using our laboratory-developed portable fNIRS system, WearLight. We designed an experimental protocol with 32 n-back WM task blocks with four different pseudo-randomized task difficulty levels. The hemodynamic response of the brain was computed from the experimental data and the evaluated brain responses due to these tasks. We observed the incremental mean hemodynamic activation induced by the increasing WM load. The left-PFC area was more activated in the WM task compared to the right-PFC. The task performance was seen to be related to the hemodynamic responses. The experimental results proved the functioning of the WearLight system in cognitive load imaging. Since the portable fNIRS system was wearable and operated wirelessly, it was possible to measure the cognitive load in the naturalistic environment, which could also lead to the development of a user-friendly brain–computer interface system.

## 1. Introduction

Human working memory (WM) is a cognitive function that has been extensively researched in cognitive psychology and cognitive neuroscience [1]. WM is defined as a short-term active memory that is responsible for the temporary storage, continuous updating, and processing of remembered information; manipulating stored information; and decision-making [2,3]. WM is essential to perform higher-order cognitive tasks and everyday activities as well. The information holding and processing capacity of the WM system is limited [1,4] and varies between individuals and tasks.

Hence, the capacity can easily reach the limit when cognitive functions perform complex tasks involving rapid storing and the processing of excessive information in a stressful environment. Different neuroimaging studies identified that neurons in the prefrontal cortex (PFC) brain areas are involved in processes necessary for WM-related tasks [5,6,7,8,9,10,11]. Working memory tasks also demonstrated activation of the bilateral network consisting of the dorsolateral prefrontal cortex (DLPFC) and ventrolateral prefrontal cortex (VLPFC), lateral premotor cortex, frontal poles, medial premotor cortices, dorsal cingulate and medial and lateral posterior parietal cortices [12].

Functional near-infrared spectroscopy (fNIRS) is a noninvasive neuroimaging technique that spatially and temporally maps hemodynamic responses as well as oxy-hemoglobin ($HbO_{2}$) and deoxy-hemoglobin (Hb) changes of the brain in order to image human brain functions [13,14,15]. Though NIRS-based measurement was first reported by Jobsis in the year 1977 [16], only in recent years has fNIRS emerged as a neuroimaging tool due to technological advancements.

The growth of fNIRS publications, future prediction, and the use of mobile fNIRS were mentioned as a new possibility “Neuroscience of the Everyday World (NEW)” [17]. However, mobile or portable fNIRS systems have many technological challenges in terms of hardware, optics, and software [18,19]. In our previous work, we discussed these challenges and attempted to address some of them by developing a wearable and configurable fNIRS system, WearLight, that can be used to study the brain in the naturalistic experimental environment [20]. In the naturalistic environment, participants can perform natural real-life activities without any restrain.

**Figure 1:**
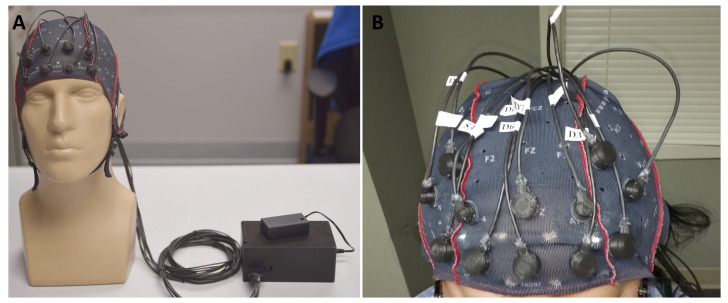
(**A**) The WearLight functional near-infrared spectroscopy (fNIRS) system. The system consists of an optode cap and a control box connected to a battery. (**B**) Participant wearing the fNIRS montage cap with four source and eight detector optodes designed for prefrontal cortex imaging in our n-back WM task.

The novelty of the presented research originates from the application of the specially designed WearLight fNIRS hardware to detect the activation of the brain regions. The WearLight (Figure 1A) offers miniaturization of the hardware with an increased sensitivity due to specifically designed source-detector (SD) pairs. The sources are LEDs with two distinct wavelengths (770 nm and 850 nm), and the detectors are highly sensitive silicon photodiodes (SiPDs) that allow placement up to 4±0.5 cm apart to create SD pairs.

3D printing technology was used to build the housing of the source and detector to comfortably place them on the head [21], and extensive studies on the performance of the device were conducted in the previous work [20]. The system was designed such that it can be used in naturalistic settings with a hassle-free setup. It is always a challenge to validate non-invasive brain monitoring technologies.

In the presented research, we aim to validate the WearLight system to measure its performance in capturing hemodynamic responses under known brain activity. Therefore, we opted for a working memory task that offers observable hemodynamic changes in the prefrontal cortex [22]. In the following sections, we will demonstrate how the WearLight fNIRS was experimented with to detect the prefrontal cortex’s activation.

## 2. Principle of fNIRS

The fNIRS sensor cap (montage) consists of multi-wavelength near-infrared (NIR) light sources and light detectors (they are commonly denoted as optodes) arranged in an experiment-specific order [23,24,25,26]. Typically, the light sources and detectors are 25–40 mm apart. The NIR light sources are sequentially turned on, and the propagated light through the tissue is absorbed by chromophores (dominantly by $HbO_{2}$ and Hb) on the cortical area of the brain [27,28,29]. The light absorption of tissues is wavelength-dependent [30]. Hence, in performing continuous spectroscopic measurements of the back-reflected diffuse light [31] from the cortical surface of the brain using the detectors attached to the montage cap, it is possible to estimate the $\Delta HbO_{2}$ and ΔHb over time [32].

With the short-separation fNIRS measurement and superficial signal regression, a more accurate brain response due to the stimulus could be recovered [21,33]. The human head has different tissue layers, such as the scalp, skull, cerebrospinal fluid, gray matter, and white matter. Each tissue type has unique optical properties and absorption ($\mu_{a}$) and reduced scattering ($\mu_{s}{}^{\prime }$) coefficient. The constituents of these and the size and shape of the tissue layers vary across humans. In diffuse optical tomography (DOT), $\mu_{a}$ and $\mu_{s}{}^{\prime }$ can be imaged by solving the inverse problem of the image reconstruction [34,35]. Simulation studies assigning $\mu_{a}$ and $\mu_{s}{}^{\prime }$ for the human head have been studied [36,37]. However, the 3D image reconstruction is computationally intensive [38] and not feasible for a portable fNIRS system [39].

In fNIRS, a simplified approach is adopted, where the scattering of photons is assumed to be rather homogeneous [40,41,42]. However, a wavelength-dependent differential pathlength factor (DPF) is incorporated in the modified Beer–Lambert law (MBLL) to account for the scattering effect and to measure only the relative changes of the concentration of $HbO_{2}$ and Hb [40,43]. A source-detector (SD) pair, sensitive to the brain activity around the midpoint of the SD pair, creates an fNIRS channel. The sensitivity (Jacobian) between a source and detector in the NIRS-based measurement for highly scattering biological tissue has a banana-like shape and strongly dependent on the SD separating distance [44,45,46,47].

## 3. Materials and Methods

### 3.1. Experimental Setup

**Figure 2:**
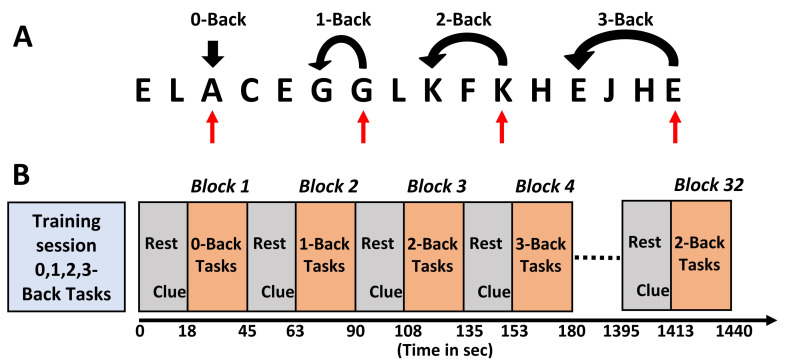
Experimental paradigm. (**A**) Concept of 0, 1, 2, and 3-back tasks, (**B**) 32 task blocks, each task block had 0, 1, 2 or 3-back conditions, and the four n-back conditions were pseudo-randomized. A complete run took about 24 min.

We recorded the hemodynamic response of the prefrontal cortex while participants performed n-back working memory task [48] using our experimental protocol graphically presented in Figure 2. Prior to the actual task, we first trained the participants on how to perform the task on a computer. The actual recording time was about 24 min as shown in the time axis in Figure 2. To record the fNIRS signal, we used a portable continuous wave fNIRS system, WearLight, developed in the University of Rhode Island, Kingston, RI, USA [20].

**Figure 3:**
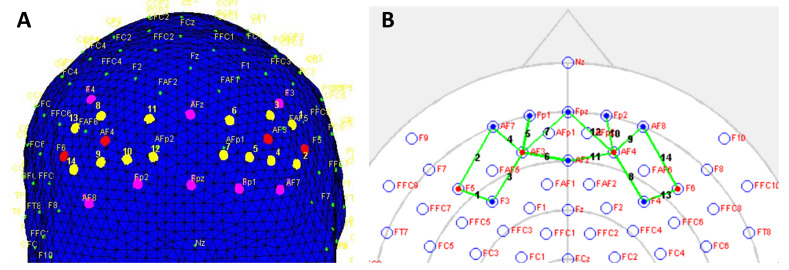
The fNIRS montage used in the experiment. (**A**) fNIRS channels (yellow label), LEDs (red), and detectors (magenta) onto a head model showing the coverage on the prefrontal areas. (**B**) LEDs (red), detectors (blue), and the fNIRS channels (green line) location on a standard 10–20 EEG system. Optode maps and figures were generated using nirsLAB software.

We designed a montage with four sources and eight detectors. Figure 3B shows the source and detector location in the standard 10–20 EEG system. The source-detector arrangement formed a total of 14 active fNIRS channels (green line in Figure 3B) covering the PFC. Figure 3A presents the fNIRS channel location on the head model. The LED light sources emit NIR light with peak wavelengths of 770 and 850 nm. The WearLight system was battery operated, and the montage cap was connected to the portable control box using a flexible cable as shown in Figure 1A. Figure 1B shows a participant wearing the fNIRS montage cap with four source optodes and eight detector optodes used in the experiment. A computer was wirelessly connected to the control box to collect fNIRS data.

We used a MATLAB-based graphical user interface (GUI) software in order to control the WearLight system and to display and store data for post-processing. The GUI software plotted the fNIRS signals in real-time that allows us for signal quality checks through visual inspection. The fNIRS signal quality varies with head size, skin tone, hair density, and thickness, and background ambient noise. Hence, for each participant, prior to recording data, we inspected the signal-to-noise ratio (SNR) by computing the coefficient of variation, the heart rate oscillations, and spikes in the signal as well as flat and saturated channels to identify the optodes that require adjustment.

We parted the hair underneath the optodes using a water-soluble gel to make a better optode–scalp interface. However, excessive application of the gel was avoided to minimize possible light channeling between the source and detector [49]. Thus, we improved the optode-scalp coupling by adjusting the optodes on the scalp to achieve the best possible fNIRS signal in a given experiment.

### 3.2. Participants

This study included 25 healthy right-handed college students as participants, aged 22–27 (mean age = 22, male = 14) and with a mean of 15 years of formal education. The participants were recruited from the student body of the University of Rhode Island, RI, USA. We advertised the study on campus through classroom announcements and flyers during an ongoing semester. The participants were informed prior to the experiment and gave written consent. In addition, the participants filled out a screening questionnaire. The inclusion criteria included no medical record of head trauma or neurological illness, no prescribed medication at the time of the study, fluency in English, and good eyesight. The study was approved by the Institutional Review Board.

### 3.3. Data Analysis

The raw experimental data collected from the brain using any fNIRS system is influenced by various artifacts, for example, physiological noise, movement artifacts, and electrical and optical noise [20,50]. Physiological signals, such as cardiac artifacts, the respiration rate, and Meyer waves, are superimposed on the fNIRS signal. Another common artifact is due to the movement of participants that alters the optode–scalp coupling resulting in spikes in the fNIRS signal.

We measured the SNR and also performed a preliminary visual inspection of the data prior to signal processing. We used molar extinction coefficient values of $HbO_{2}$ and Hb corresponding to wavelengths of 770 and 850 nm, respectively, from the literature. The MBLL was applied to convert the data from the voltage (mV) to the relative concentration change for $\Delta HbO_{2}$ and ΔHb. We processed the raw fNIRS data using a traditional fNIRS signal processing pipeline on our MATLAB-based fNIRS data processing software to retain the noise-free low-frequency fNIRS signal (0.01 and 0.2 Hz).

**Figure 4:**
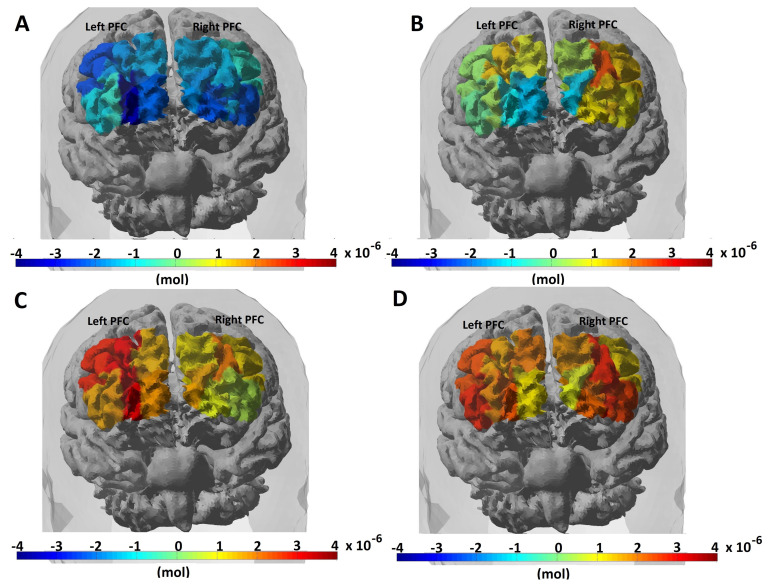
Increasing oxyhemoglobin ($HbO_{2}$) response with difficulty levels in the n-back task. (**A**) 0-back, (**B**) 1-back, (**C**) 2-back, and (**D**) 3-back.

We also spatially mapped the fNIRS channels (Figure 3) on the brain for the topographic visualization [Figure 4]. The time series $\Delta HbO_{2}$ and ΔHb were segmented into −5- to 40-s epochs (5-s pre-stimulus, 27-s stimulus, and 13-s post-stimulus) with respect to the onset of the task condition. The 5-s pre-stimulus duration was used for the baseline normalization of each task block. We performed channel-wise block averaging for the four task conditions to visualize and analyze task-induced hemodynamic response variation over all the fNIRS channels [51]. The block averaging was performed by averaging the hemodynamic response over the blocks of the same stimulus condition over all the participants.

## 4. Results

### 4.1. Task Performance

We analyzed the participants’ task performance data to verify if the participants experienced different difficulty levels in the four n-back conditions. We also conducted a feedback session after each experiment to note the participants’ experiences. We evaluated the number of wrong reactions when participants incorrectly identified a non-target letter as a target by pressing the “0” key. The missing reaction is when participants failed to respond by pressing the “0” key when a target stimulus was presented. The wrong reaction and accuracy do not include the missing targets.

**Figure 5:**
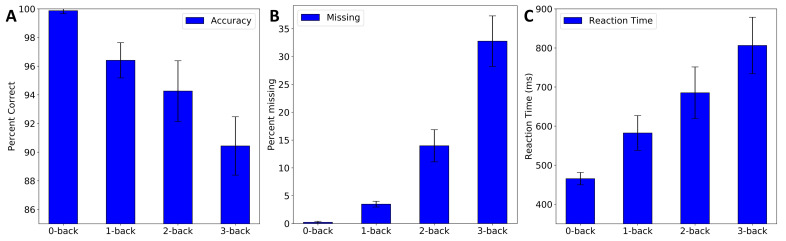
Task performance. Average (**A**) % correct, (**B**) % missing, and (**C**) reaction times. Whiskers show the standard deviations between subjects.

As seen in Figure 5, the task performance in terms of the accuracy and missing and reaction times, varied across the task conditions. On average, the task performance decreased with the order of n-back task as seen in Figure 5. The accuracy for 0, 1, 2, and 3-back tasks were on average 99.86±0.2%, 96.4±1.22%, 94.26±2.11%, and 90.43±2.03%, respectively. There were 0.22±0.2% targets missed by the participants in the 0-back task.

The percentage of targets missed increased from 3.48±0.25% for the 1-back to 13.97±2.8% for the 2-back to 32.76±4.53% for the 3-back task. The reaction time for 0, 1, 2, and 3-back tasks were on average 465±15, 582±44, 685±66 and 806±72 ms respectively. From three separate one-way ANOVA tests, we also found that the accuracy and missing and reaction times were significantly different in the four task conditions (each p<0.001). From a multiple pairwise post hoc comparison analysis using the Tukey HSD test, we found that all the pairs were significantly different (p<0.001).

We observed decreased accuracy and increased missing and reaction times with *n* as seen in Figure 5. This shows that, on average, the participants experienced the task difficulty linearly. The participants were from the Bachelor of Science program, and they went through a short n-back task training session prior to the actual session. The training session included a verbal illustration of the task and a short practice session with a completely different n-back test set to teach how to perform the n-back task on the computer.

Including other factors, such as the participants’ academic background, age, and ongoing academic activities, the training might have resulted in a better participant engagement level and task performance. However, differences in targets missed, wrong reactions, and reaction times in the 0, 1, 2, and 3-back task conditions were noticed. Analyzing the task performance and subjective data in the feedback session, we clearly verified that the four n-back task conditions had significantly different difficulty levels, and this demanded four different mental workload levels.

### 4.2. Brain Hemodynamic Response

The data analysis was performed after the raw data were converted to oxy-hemoglobin ($HbO_{2}$) and deoxy-hemoglobin (Hb) changes using the data analysis strategy explained in Section 3.3. Figure 4 shows topographic images of the mean $HbO_{2}$ values in the four n-back tasks of a participant. We observed that $HbO_{2}$ variation over both the hemispheres in the PFC and the $HbO_{2}$ response increased with the difficulty level of the n-back task. After obtaining the time course of the block-averaged hemodynamic response of all the participants, we evaluated the mean response over all the participants to identify areas on the PFC that showed, on average, a greater hemodynamic response and WM load levels due to the n-back task stimulation.

Table 1 presents the average $HbO_{2}$ in left PFC channels (Ch 1–Ch 7) and right PFC channels (Ch 8–Ch 14) for the 1-back, 2-back, and 3-back task conditions. However, from this single averaged value, it is unclear how $HbO_{2}$ and Hb changed in the task block. A more comprehensive way is to visualize the time course of the $HbO_{2}$ and Hb in response to the stimulus to understand the trend. Hence, we present the channel-wise hemodynamic response during the entire course of the task condition rather than only presenting the average single value.

**Figure 6:**
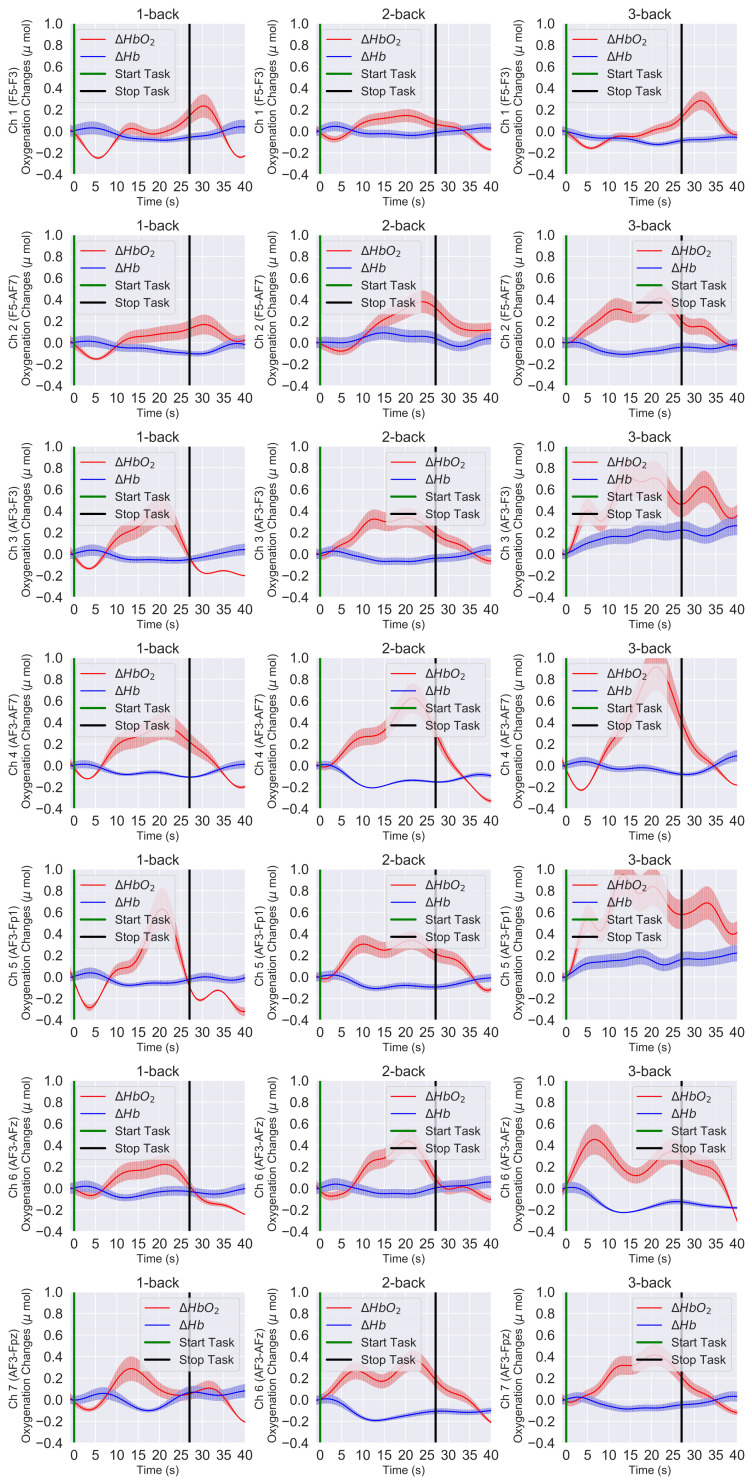
The left PFC block-averaged time trace, and the channel-wise mean $HbO_{2}$ and Hb responses of all 25 participants at the group level. $HbO_{2}$ (solid red lines), Hb (solid blue lines), and the error bars correspond to the ± SEM (standard error of means). Vertical green and black lines are the start and stop markers for the task.

**Figure 7:**
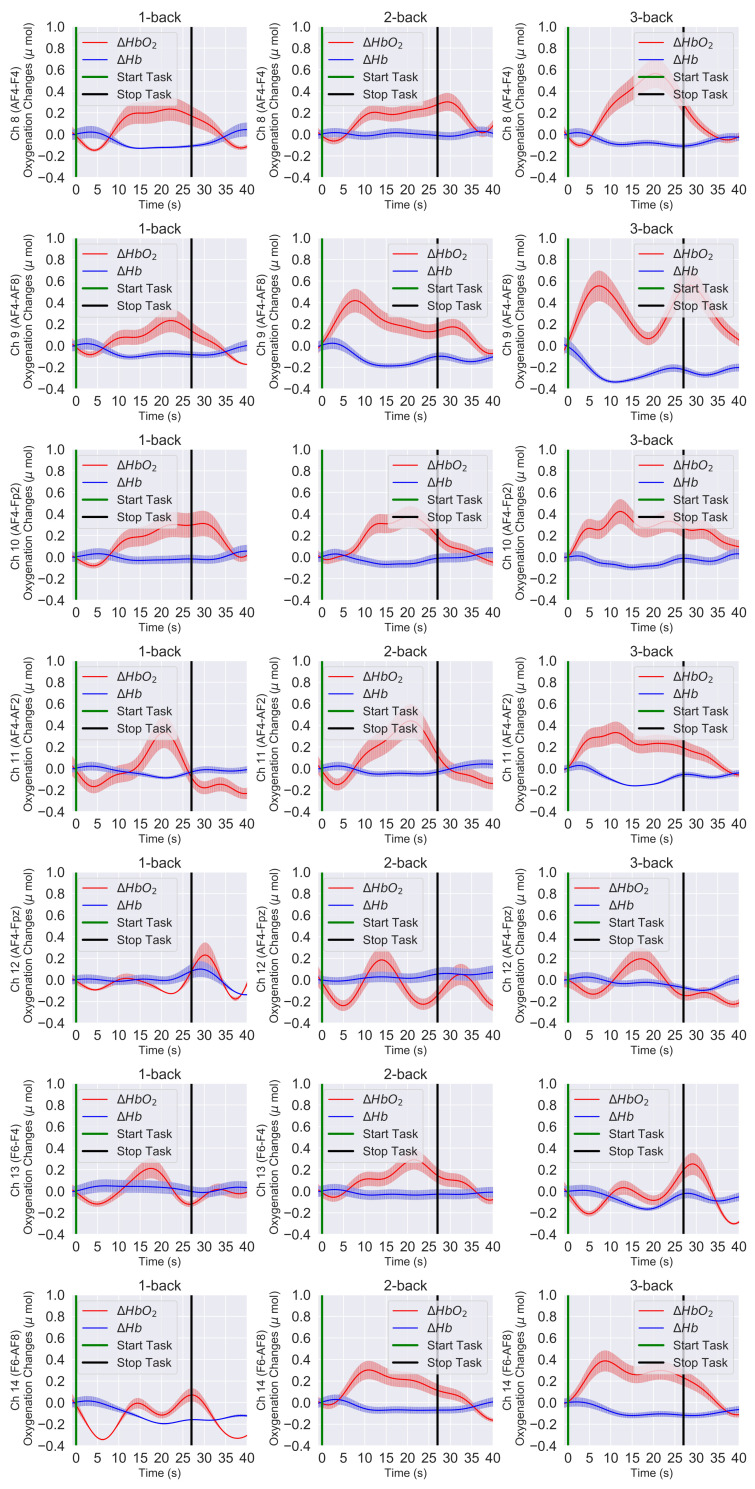
The right PFC block-averaged time trace, and the channel-wise mean $HbO_{2}$ and Hb responses of all 25 participants at the group level. $HbO_{2}$ (solid red lines), Hb (solid blue lines), and the error bars correspond to the ±SEM (standard error of means). Vertical green and black lines are the start and stop markers for the task.

Figure 6 and Figure 7 present the time trace of the block averaging results and the channel-wise mean $HbO_{2}$ and Hb responses at the group level for the 1-back, 2-back, and 3-back conditions. In our case, block averaging refers to the average over the blocks of the same task condition and the participants overall. Figure 6 shows all the left PFC channels (Ch 1 to Ch 7), and Figure 7 shows all the right PFC channels (Ch 8 to Ch 14). If we compare Figure 6 and Figure 7, we see that, in both the left and right PFC, the $HbO_{2}$ level reached the peak in the 3-back condition and demonstrated an overall lower response in the 2-back condition, whereas in the 1-back condition, it was minimum.

In the 0-back condition, the response was close to the baseline level and, hence, not presented in the figures. On the other hand, we noticed that the Hb response was the opposite that of the $HbO_{2}$ response (Figure 6 and Figure 7). The Hb response slightly decreased with the increase of $HbO_{2}$. We also observed consistent hemodynamic activities over both the left and right hemispheres. Regarding the channel location, the yellow label in Figure 3A and green line in Figure 3B on the standard 10–20 EEG system can be seen on the PFC area of the brain. Hence, from the spatial distribution of the channels, we observed a slightly higher left PFC (Ch 3–Ch 5) (Figure 6 row 3 to 5) activation compared with the right PFC (Figure 7 row 3 to 50).

**Figure 8:**
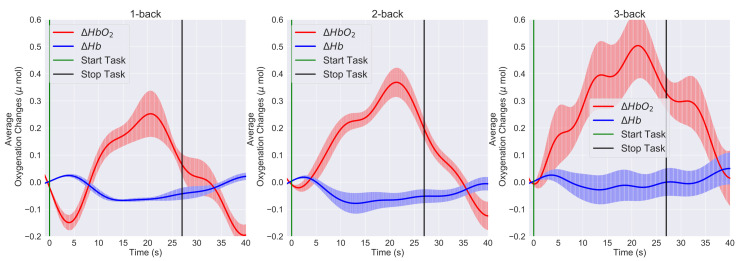
The left PFC grand block-averaging result in the 1-back, 2-back, and 3-back conditions. $HbO_{2}$ (solid red lines), Hb (solid blue lines), and the error bars correspond to the ± SEM (standard error of means). Vertical green and black lines are the start and stop markers for the task.

**Figure 9:**
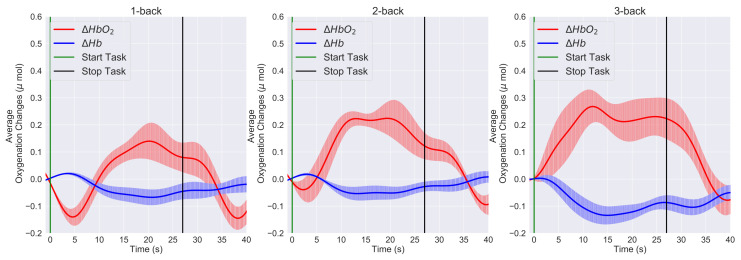
The right PFC grand block-averaging result in the 1-back, 2-back, and 3-back conditions. $HbO_{2}$ (solid red lines), Hb (solid blue lines), and the error bars correspond to the ± SEM (standard error of means). Vertical green and black lines are the start and stop markers for the task.

Figure 8 shows the left PFC grand block-averaging result, averaged over the blocks of the same task conditions and the overall left channels and participants, for the 1-back, 2-back, and 3-back conditions. Similarly, Figure 9 shows the right PFC grand block averaging result. The differences between the left and right PFC hemodynamic response can be further visualized by comparing the grand block-averaging results (Figure 8 and Figure 9) and checking the data in Table 1.

We also performed two-way ANOVA for the repeated measures (Four task conditions x 14 channels). The two-way ANOVA test showed a significant main effect for the channel (p<0.001). This can be interpreted as higher hemodynamic activity in certain channels irrespective of the task condition (p<0.001). Further from the post hoc analysis, we found that channels 3, 4, and 5 had higher hemodynamic activity in all the task conditions (p<0.05). The significant interactions between task condition and channel (p<0.001) are also indicative of hemodynamic activity due to the WM load.

## 5. Discussion and Conclusions

Many neuroimaging studies provided evidence that the prefrontal cortex was gradually activated more when the working memory load was increased. Our results, the quantitative comparison of the hemodynamic responses of the brain in the 1-back, 2-back, and 3-back task conditions (Figure 6 and Figure 7) and statistical significance level (p<0.001), confirmed that the higher PFC activation was due to the higher difficulty level of the WM task.

Both hemispheres showed the same trend; however, a slightly stronger activation was observed over the left cortex and channels 3, 4, and 5 (p<0.05). The difference between left and right PFC was also noticed in the grand block averaging results, Figure 8 and Figure 9. The participants’ task performance was evaluated using the accuracy and missing and reaction times. Figure 5 shows that, on average, the experimental protocols clearly induced four different task difficulty levels to the participants. These differences were statistically significant (p<0.001).

The presented investigation by our WearLight fNIRS system supports the results from other studies [14,24,52], showing a stronger hemodynamic response with incremental WM task difficulty. While there is a strong interest in understanding the brain through noninvasive technologies, we believe that the validation study of our WearLight fNIRS takes us one step forward. In the future, we aim to focus on enhancing the hardware of the WearLight fNIRS to improve the spatial resolution that could allow a deeper understanding of the brain’s hemodynamic activities and their association with brain activation.

In conclusion, wearable fNIRS is a promising technology for noninvasive brain imaging in the naturalistic environment that can lead to the development of widely applicable Brain–Computer Interface (BCI) applications, such as vehicle navigation simulations, virtual reality exercises, surgery training, classroom environments, and medical rehabilitation. The fNIRS-based BCI may require integration with EEG to overcome the limitations of temporal resolution [14]. Physiological sensing, such as heart rate variability and respiration rate monitoring, can further enhance the accuracy of such hybrid BCI systems [53].

## Figures and Tables

**Table 1:** The average $HbO_{2}$ in the left PFC channels (Ch 1–Ch 7) and right PFC channels (Ch 8–Ch 14) for 1-back, 2-back, and 3-back task condition.

Channel	Source-Detector	1-Back	2-Back	3-Back
Ch 1	F5-F3	−0.02	0.04	0.02
Ch 2	F5-AF7	0.04	0.14	0.18
Ch 3	AF3-F3	0.05	0.16	0.46
Ch 4	AF3-AF7	0.11	0.17	0.23
Ch 5	AF3-Fp1	0.03	0.16	0.56
Ch 6	AF3-AFz	0.02	0.12	0.19
Ch 7	AF3-FPz	0.06	0.13	0.16
Ch 8	AF4-F4	0.07	0.14	0.19
Ch 9	AF4-AF8	0.05	0.15	0.27
Ch 10	AF4-Fp2	0.14	0.15	0.24
Ch 11	AF4-AF2	−0.02	0.09	0.16
Ch 12	AF4-Fpz	−0.02	−0.05	−0.05
Ch 13	F6-F4	0.01	0.09	−0.03
Ch 14	F6-AF8	−0.11	0.11	0.17

## Data Availability

The data are restricted from public availability, as they contain confidential information that may conflict with the privacy of the research participants.

## References

[1] Baddeley A. (2003). Working memory: Looking back and looking forward. Nat. Rev. Neurosci..

[2] Fuster J.M. (1995). Memory in the Cerebral Cortex: An Empirical Approach to Neural Networks in the Human and Nonhuman Primate.

[3] Goldman-Rakic P.S. (1995). Architecture of the Prefrontal Cortex and the Central Executive. Structure and Functions of the Human Prefrontal Cortex.

[4] Miller G.A. (1956). The magical number seven, plus or minus two: Some limits on our capacity for processing information. Psychol. Rev..

[5] D’Esposito M., Postle B.R., Ballard D., Lease J. (1999). Maintenance versus Manipulation of Information Held in Working Memory: An Event-Related fMRI Study. Brain Cogn..

[6] Spitzer B., Goltz D., Wacker E., Auksztulewicz R., Blankenburg F. (2014). Maintenance and manipulation of somatosensory information in ventrolateral prefrontal cortex. Hum. Brain Mapp..

[7] Smith E.E., Jonides J. (1997). Working Memory: A View from Neuroimaging. Cogn. Psychol..

[8] Klingberg T. (2006). Development of a superior frontal–intraparietal network for visuo-spatial working memory. Neuropsychologia.

[9] Funahashi S. (2006). Prefrontal cortex and working memory processes. Neuroscience.

[10] Pessoa L., Ungerleider L.G. (2004). Top-Down Mechanisms for Working Memory and Attentional Processes. The Cognitive Neurosciences.

[11] Pereira T., Castro M.A., Villafaina S., Carvalho Santos A., Fuentes-García J.P. (2020). Dynamics of the Prefrontal Cortex during Chess-Based Problem-Solving Tasks in Competition-Experienced Chess Players: An fNIR Study. Sensors.

[12] Funahashi S., Bruce C.J., Goldman-Rakic P.S. (1989). Mnemonic coding of visual space in the monkey’s dorsolateral prefrontal cortex. J. Neurophysiol..

[13] Steinbrink J., Villringer A., Kempf F., Haux D., Boden S., Obrig H. (2006). Illuminating the BOLD signal: Combined fMRI–fNIRS studies. Magn. Reson. Imaging.

[14] Aghajani H., Garbey M., Omurtag A. (2017). Measuring Mental Workload with EEG+fNIRS. Front. Hum. Neurosci..

[15] Chen W.L., Wagner J., Heugel N., Sugar J., Lee Y.W., Conant L., Malloy M., Heffernan J., Quirk B., Zinos A. (2020). Functional Near-Infrared Spectroscopy and Its Clinical Application in the Field of Neuroscience: Advances and Future Directions. Front. Neurosci..

[16] Jöbsis F.F. (1977). Noninvasive, Infrared Monitoring of Cerebral and Myocardial Oxygen Sufficiency and Circulatory Parameters. Science.

[17] von Lühmann A., Zheng Y., Ortega-Martinez A., Kiran S., Somers D.C., Cronin-Golomb A., Awad L.N., Ellis T.D., Boas D.A., Yücel M.A. (2021). Towards Neuroscience of the Everyday World (NEW) using functional Near Infrared Spectroscopy. Curr. Opin. Biomed. Eng..

[18] Pinti P., Aichelburg C., Gilbert S., Hamilton A., Hirsch J., Burgess P., Tachtsidis I. (2018). A Review on the Use of Wearable Functional Near-Infrared Spectroscopy in Naturalistic Environments. Jpn. Psychol. Res..

[19] Saikia M.J. (2021). Internet of things-based functional near-infrared spectroscopy headband for mental workload assessment. Optical Techniques in Neurosurgery, Neurophotonics, and Optogenetics.

[20] Saikia M., Besio W., Mankodiya K. (2019). WearLight: Toward a Wearable, Configurable Functional NIR Spectroscopy System for Noninvasive Neuroimaging. IEEE Trans. Biomed. Circuits Syst..

[21] Saikia M., Mankodiya K. (2019). 3D-printed human-centered design of fNIRS optode for the portable neuroimaging. Progress in Biomedical Optics and Imaging—Proceedings of SPIE.

[22] Blokland G.A., McMahon K.L., Hoffman J., Zhu G., Meredith M., Martin N.G., Thompson P.M., de Zubicaray G.I., Wright M.J. (2008). Quantifying the heritability of task-related brain activation and performance during the N-back working memory task: A twin fMRI study. Biol. Psychol..

[23] Siddiquee M.R., Atri R., Marquez J.S., Hasan S.M.S., Ramon R., Bai O. (2020). Sensor Location Optimization of Wireless Wearable fNIRS System for Cognitive Workload Monitoring Using a Data-Driven Approach for Improved Wearability. Sensors.

[24] Meidenbauer K.L., Choe K.W., Cardenas-Iniguez C., Huppert T.J., Berman M.G. (2021). Load-dependent relationships between frontal fNIRS activity and performance: A data-driven PLS approach. NeuroImage.

[25] Saikia M.J., Kuanar S., Borthakur D., Vinti M., Tendhar T. (2021). A Machine Learning Approach to Classify Working Memory Load from Optical Neuroimaging Data.

[26] Fernandez Rojas R., Liao M., Romero J., Huang X., Ou K.L. (2019). Cortical Network Response to Acupuncture and the Effect of the Hegu Point: An fNIRS Study. Sensors.

[27] Baker J.M., Bruno J.L., Gundran A., Hosseini S.M.H., Reiss A.L. (2018). fNIRS measurement of cortical activation and functional connectivity during a visuospatial working memory task. PLoS ONE.

[28] Wolf M., Ferrari M., Quaresima V. (2007). Progress of near-infrared spectroscopy and topography for brain and muscle clinical applications. J. Biomed. Opt..

[29] Saikia M.J., Brunyé T.T. (2021). K-means clustering for unsupervised participant grouping from fNIRS brain signal in working memory task. Optical Techniques in Neurosurgery, Neurophotonics, and Optogenetics.

[30] Saikia M.J. (2021). An embedded system based digital onboard hardware calibration for low-cost functional diffuse optical tomography system. Optics and Biophotonics in Low-Resource Settings VII.

[31] Saikia M.J., Kanhirodan R. (2019). Development of handheld near-infrared spectroscopic medical imaging system. Proceedings of the Biophotonics Congress: Optics in the Life Sciences Congress 2019 (BODA,BRAIN,NTM,OMA,OMP).

[32] Saikia M., Mankodiya K., Kanhirodan R. (2019). A point-of-care handheld region-of-interest (ROI) 3D functional diffuse optical tomography (fDOT) system. Progress in Biomedical Optics and Imaging—Proceedings of SPIE.

[33] Saikia M.J., Manjappa R., Mankodiya K., Kanhirodan R. (2018). Depth sensitivity improvement of region-of-interest diffuse optical tomography from superficial signal regression. Optics InfoBase Conference Papers.

[34] Poorna R., Kanhirodan R., Saikia M.J., Fantini S., Taroni P. (2021). Square-waves for frequency multiplexing for fully parallel 3D diffuse optical tomography measurement. Optical Tomography and Spectroscopy of Tissue XIV.

[35] Saikia M.J. (2021). A spectroscopic diffuse optical tomography system for the continuous 3D functional imaging of tissue -a phantom study. IEEE Trans. Instrum. Meas..

[36] Saikia M., Kanhirodan R. High performance single and multi-GPU acceleration for Diffuse Optical Tomography. Proceedings of the 2014 International Conference on Contemporary Computing and Informatics, IC3I 2014.

[37] Doulgerakis M., Eggebrecht A., Wojtkiewicz S., Culver J., Dehghani H. (2017). Toward real-time diffuse optical tomography: Accelerating light propagation modeling employing parallel computing on GPU and CPU. J. Biomed. Opt..

[38] Saikia M.J., Kanhirodan R., Mohan Vasu R. (2014). High-speed GPU-based fully three-dimensional diffuse optical tomographic system. Int. J. Biomed. Imaging.

[39] Saikia M., Manjappa R., Kanhirodan R. (2017). A cost-effective LED and photodetector based fast direct 3D diffuse optical imaging system. Optics InfoBase Conference Papers.

[40] Delpy D.T., Cope M., van der Zee P., Arridge S., Wray S., Wyatt J. (1988). Estimation of optical pathlength through tissue from direct time of flight measurement. Phys. Med. Biol..

[41] Huppert T.J., Diamond S.G., Franceschini M.A., Boas D.A. (2009). HomER: A review of time-series analysis methods for near-infrared spectroscopy of the brain. Appl. Opt..

[42] Sassaroli A., Fantini S. (2004). Comment on the modified Beer-Lambert law for scattering media. Phys. Med. Biol..

[43] Chiarelli A.M., Perpetuini D., Filippini C., Cardone D., Merla A. (2019). Differential pathlength factor in continuous wave functional near-infrared spectroscopy: Reducing hemoglobin’s cross talk in high-density recordings. Neurophotonics.

[44] Saikia M.J., Kanhirodan R. (2016). Region-of-interest diffuse optical tomography system. Rev. Sci. Instruments.

[45] Doulgerakis M., Eggebrecht A.T., Dehghani H. (2019). High-density functional diffuse optical tomography based on frequency-domain measurements improves image quality and spatial resolution. Neurophotonics.

[46] Saikia M.J. (2021). Design and development of a functional diffuse optical tomography probe for real-time 3D imaging of tissue. Optical Tomography and Spectroscopy of Tissue XIV.

[47] Saikia M.J., Kanhirodan R. (2014). Development of DOT system for ROI scanning. Proceedings of the International Conference on Fibre Optics and Photonics.

[48] Owen A.M., McMillan K.M., Laird A.R., Bullmore E. (2005). N-back working memory paradigm: A meta-analysis of normative functional neuroimaging studies. Hum. Brain Mapp..

[49] Sieno L.D., Contini D., Presti G.L., Cortese L., Mateo T., Rosinski B., Venturini E., Panizza P., Mora M., Aranda G. (2019). Systematic study of the effect of ultrasound gel on the performances of time-domain diffuse optics and diffuse correlation spectroscopy. Biomed. Opt. Express.

[50] Lee G., Jin S.H., An J. (2018). Motion Artifact Correction of Multi-Measured Functional Near-Infrared Spectroscopy Signals Based on Signal Reconstruction Using an Artificial Neural Network. Sensors.

[51] Santosa H., Fishburn F., Zhai X., Huppert T.J. (2019). Investigation of the sensitivity-specificity of canonical- and deconvolution-based linear models in evoked functional near-infrared spectroscopy. Neurophotonics.

[52] Kumar V., Nichenmetla S., Chhabra H., Sreeraj V.S., Rao N.P., Kesavan M., Varambally S., Venkatasubramanian G., Gangadhar B.N. (2021). Prefrontal cortex activation during working memory task in schizophrenia: A fNIRS study. Asian J. Psychiatry.

[53] Liu Y., Ayaz H., Shewokis P.A. (2017). Multisubject “Learning” for Mental Workload Classification Using Concurrent EEG, fNIRS, and Physiological Measures. Front. Hum. Neurosci..

